# Supplementary material for: Centralized softening as a solution to chloride pollution: An empirical analysis based on Minnesota cities
Source: PLoS One. 2021 Feb 5;16(2):e0246688. doi: 10.1371/journal.pone.0246688 (PMC7864447; doi:10.1371/journal.pone.0246688)
Supplement: Attachment — Submitted filename: Response to Reviewers.docx [file pone.0246688.s003.docx]

# Response to Reviewers for manuscript titled “Centralized softening as a solution to chloride pollution: An empirical analysis based on Minnesota cities

*January 22, 2021*

Dear editor and reviewers,

Thank you for reviewing our manuscript and for your helpful comments. This rebuttal letter contains our responses to each of the comments from the reviewers (part 6 of the letter to authors from Plos-One) for the second revision of the manuscript. Please let us know if you have any question/s.

Sincerely,

Baishali Bakshi (on behalf of the author team)

6. Review Comments to the Author

Reviewer #1: (No Response)

Reviewer #2: The discussion on pages 6-7 adequately addresses my concern about the national applicability of this case study, but it should come earlier in the manuscript introduction and be associated with Figure 2--i.e., the paper should move from a national-level discussion of the problem to the Minnesota case study so that the case study is adequately framed from the beginning. The current back-and-forth between Minnesota and national considerations in the Introduction is jarring. It also might be beneficial to circle back to the national implications at the end of the paper.

Response: We agree and have reorganized the content of the introduction so that the national context discussion now precedes and leads to the discussion on the Minnesota case study. This reorganization has led to changes in the position of Figures 1 and 2: now Figure 2 is actually Figure 1 and vice versa. We have also updated the numbering of the in-text citations and synced them in the references. Finally, we have added a sentence in the conclusion that circles back to the national implications of the problem discussed in the introduction.

Reviewer #3: This is ready. A couple things caught when re-reading:
In the abstract lime shouldn't be capitalized
ll76 I think you mean reduction of Cl
pg 17, last paragraph. To say the hardness is the same is too definite. Similar would be better (e.g., lawn irrigation would make the output higher.

Response: Thank you. We have made the following changes based on your comments.

a. We have un-capitalized lime in the abstract.
b. Here is the sentence from lines 76-77: ‘‘Chloride in WWTP discharges is generally viewed as infeasible to treat due to high cost and limited availability of treatment technologies”.

We prefer the term ‘remove’ to replace the term ‘treat’ because ‘removing’ is a more specific term from the perspective of an engineer. Removing means taking chloride out of the wastewater. The result of “removing” is a reduction in chloride but the converse isn’t true. Based on your comment and the above, we have revised the sentence as:

“Removing chloride from WWTP discharges is infeasible due to high cost and limited availability of treatment technologies”, in the revised manuscript.

c. We have replaced ‘the same’ with ‘similar’ in the last paragraph of page 17 in the revised manuscript.

Note: Apart from these changes, we have made some additional changes to correct typos, add more references, and add more updated/relevant content. These additional changes are listed below.

1. We un-capitalized ‘Lime’ in Lime-softening throughout the manuscript.
2. We updated reference #16 as the corresponding manuscript has just been published.
3. We corrected a typo (crossed out Effluent Limits Unit) from the affiliation of the corresponding author.
4. We added the word ‘sources’ to sentence 2 (line 29) in the abstract.
5. We added the word ‘statewide’ to line 389, page 16 in the Revised Manuscript with Track Changes.
6. We explained corrosion control and operator costs as part of O&M costs of CS technologies, on page 24 and referred to these in the ‘Factors considered’ sub-section on page 33 of the Revised Manuscript with Track Changes.
7. We added 6 additional references, as required by the revision and those excluded by oversight earlier.
8. We acknowledged our sister agency the Minnesota Department of Health in the acknowledgment section, which we had omitted before owing to oversight.
